# Floristic composition and utilization of ethnomedicinal plant species in home gardens of the Eastern Himalaya

**DOI:** 10.1186/s13002-019-0293-4

**Published:** 2019-02-19

**Authors:** Nazir A. Pala, Biplov C. Sarkar, Gopal Shukla, Nakul Chettri, Shovik Deb, Jahangeer A. Bhat, Sumit Chakravarty

**Affiliations:** 1 Department of Forestry, Uttar Banga Krishi Viswavidyalaya, Pundibari, WB India; 2 International Centre for Integrated Mountain Development (ICIMOD), GPO Box 3226, Kathmandu, Nepal; 3 Department of Soil Science and Agricultural Chemistry, Uttar Banga Krishi Viswavidyalaya, Pundibari, WB India; 4 School of Agricultural Sciences and Forestry, College of Agriculture, Fisheries and Forestry, Fiji National University, Koronivia, PO Box 1544, Nausori, Fiji Islands

**Keywords:** Indigenous, Healthcare, Culture, Dominant, Families, Disease

## Abstract

**Background:**

Home gardens are popular micro land-use system and are socioeconomically linked with people for their livelihood. In the foothill region of Eastern Himalaya, very less documentations are available on species richness of the home gardens, particularly on the ethnomedicinal plants. We assumed that the home garden owners of the study site are domesticating ethnomedicinal plants which are not easily accessible to them in the wild due to distant forest. This study was planned to explore and document the diversity and population status of ethnomedicinal plants in the home gardens along with its ethnomedicinal use.

**Methods:**

The present study was conducted in the home gardens of Cooch Behar district of West Bengal from May 2017 to May 2018. A multidisciplinary approach like collection of plant specimen, interview with structured questionnaire for documenting the utilization pattern, and quadrat methods for population study was applied. We selected 150 study sites randomly in the village cluster. The owners of the gardens were the respondents for the household survey. The study documented diversity, population size, and medicinal uses of ethnomedicinal plant species identified by the garden owners growing or being grown in their gardens.

**Results:**

A total of 260 plant species were reported, of which, 53 were utilized for different ethnomedicinal applications. These 53 species were represented by 35 families and 45 genera. Most of these ethnomedicinal species were woody perennials (37.73%). *Cocus nucifera* dominated the list with highest number of use followed by *Hibiscus rosa-sinensis.* The use value of the species varied from 0.006 to 0.53, while the fidelity value (%) ranged from 2.29 to 93.75%. The leaves of the plants were mostly used for ethnomedicinal applications (19 species) followed by fruits (12 species) and bark (9 species), and the least was the root (7 species). We documented 20 different ailments/diseases cured by using these plants. In some cases, more than one species are used to cure a disease or ailment. As many as 10 species were used to cure only stomach-related problems. Some more diseases like cough and cold and jaundice were treated using six and four species, respectively.

**Conclusion:**

This documented list of 260 plant species including 53 ethnomedicinal ones from the home gardens of the study area indicates that these gardens are key in maintaining diversity and source of healthcare system in agricultural dominant landscape. Documenting such ecological status and traditional applications becomes a prerequisite for developing conservation and management strategies of home gardens to be included in the mainstream conservation processes.

**Electronic supplementary material:**

The online version of this article (10.1186/s13002-019-0293-4) contains supplementary material, which is available to authorized users.

## Background

Home gardens are basic production units contributing to social and cultural well-being in rural areas. These units are becoming dominant and promising land-use system in many part of the tropics that maintain high levels of diversity, productivity, and sustainability endowed with important ecosystem functions [1, 2]. Home gardens have been documented as sources of a diverse and stable supply of goods and services [3–5]. The realization that the home gardens are also a vital reservoir of unique genetic diversity including the ethnomedicinal plants has recently led to more careful research to understand the role of home gardens as in situ genetic diversity [1, 6–11]. The opportunity of using home garden as means for conservation of crop and forest plant genetic diversity as an effective complementary measure to ex situ strategies has been reported [12]. Such traditional knowledge involved in home gardens is not only a cultural heritage but might be highly valuable for many purposes, for instance, to secure the sustainability of gardening or to conserve globally significant agro and medicinal plant diversity [3, 13–15]. Many traditional crop species were reported to have medicinal properties which are retained in the traditional knowledge of the local people through home gardens [16]. Medicinal plants in home gardens are either deliberately cultivated or come up spontaneously as wild and weedy species [2, 17]; they are identified as one of the key characteristics of traditional home gardens [3, 18]. They have played a major role in maintaining primary and basic healthcare of rural communities from time immemorial [19, 20].

On account of high rainfall, rich ethnic diversity, and biodiversity, Cooch Behar district, located in the foothill of North Bengal, has been witnessing a high rate of adoption of home gardens as in other humid areas [1]. Home gardens of Cooch Behar are smaller with an average size of 0.61 ha than that of other parts of India or elsewhere but are generally high in native diversity including ethnomedicinal plants [1, 7–11]. In India, particularly in West Bengal, people living in remote and rural areas are still dependent on traditional medicines for the treatment of various ailments due to lack of modern medical facilities and poor socioeconomic conditions [21–27]. Further, the elders of the region are skeptical on the retention of traditional knowledge and advocate documentation of these old age traditions and domestication of such valuable plant species for renewed interests among new generations and cultural transmissions of this valuable knowledge system [14–29]. Since traditional knowledge on ethnobotany is being eroded through modernization along with loss of plant biodiversity, documentation of this knowledge is crucial for safeguarding and preserving for future generations [28–30].

One of the pioneering studies on ethnobotanical study in home gardens reported only 17 species [22]. The number increased to 78 species after a decade [27, 31]. This indicates increasing use and importance given by the local community residing in the area. Both of these studies reported that the ethnobotanical plant species cultivated have actually been grown in their home garden and contributing to the conservation of the species for domestic use. Similar studies on home gardens maintaining rich biodiversity of ethnobotanical plants were also reported from Ethiopia [32–36]. This clearly indicates the increase in community consciousness on the conservation values of these ethnobotanical species. The practice also ensures sustainable harnessing of the valuable resource through domestication and leaving the species intact in the wild.

Raising home gardens by local people according to [37] is basically “learning to live with change and uncertainty”. The Cooch Behar district has only about 10% of its total geographical area under forest as compared to about 45% and 60% forest cover of the total geographical area in the adjacent districts of Jalpaiguri and Darjeeling, respectively [38]. We therefore expected that the garden owners of Cooch Behar district might be using and conserving the ethnomedicinal plants required for sustaining their primary nutritional care and healthcare by growing and maintaining in their own garden as there is less forest accessible to them or might be distantly located [39–44]. The national government is also now promoting and integrating the use of traditional medicines in the national healthcare through establishing a separate Ministry of Ayush. In spite of all this, the urban population accepts a new wild plant product only after a proper testimony of the specialists [27]. Though home gardens are a very common feature of this region, very limited efforts have been made for the documentation of one of its key characteristics, i.e., ethnomedicinal plants [8–10]. We explored and documented the diversity and population status of ethnomedicinal plants in the home gardens of Cooch Behar along with its associated traditional knowledge and use value. The information and data generated from this study can form a basis for conservation and sustainable utilization of ethnomedicinal plants and also contribute to preserve cultural and genetic diversity.

## Materials and methods

### Study area

**Fig. 1:**
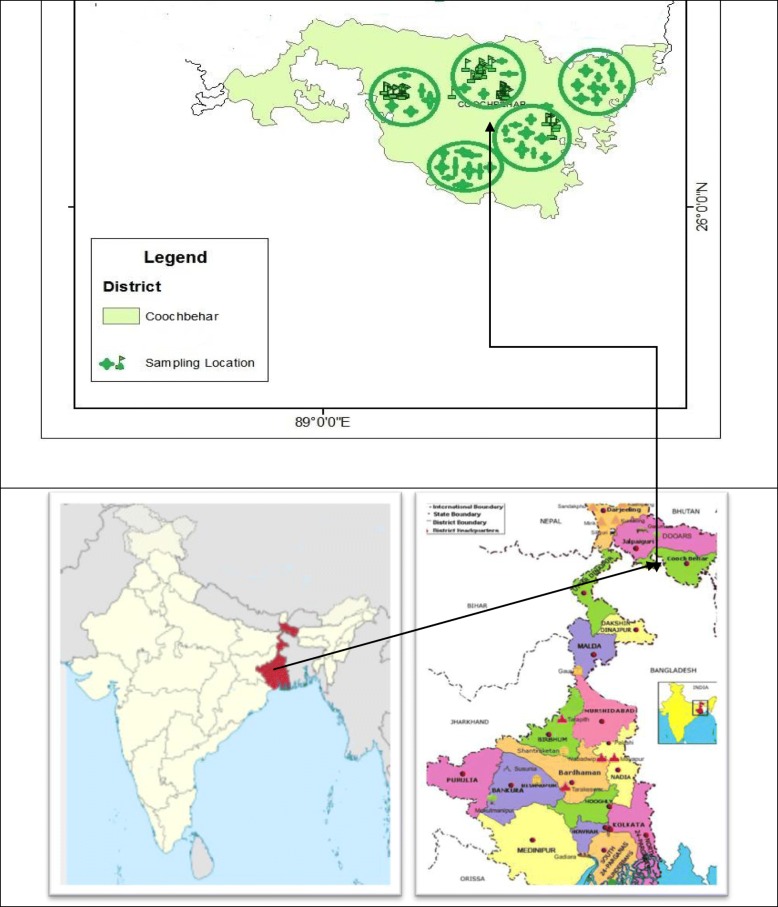
Map of the study area showing home garden locations

The present study was carried in Cooch Behar district of West Bengal, India. The area is located in the Terai region at the northeastern part of West Bengal surrounded by the district of Jalpaiguri and Alipurduar to the north and west (Fig. 1). The area also shares state boundary with Assam to the east and international boundary with Bangladesh at south, southeast, and southwest. The district is located between 260 32′ 20″ N to 250 57′ 40″ N latitude and 89° 54′ 35″ E to 88° 47′ 40″ E longitude with an average altitude of 43 msl. Cooch Behar is characterized by humid weather and abundant rain. There is a considerable variation in the seasonal and diurnal temperature. In general, July is the hottest month while January is the coldest one. The average minimum and maximum temperature varied from 23.08 °C during winter (January) to 33.42 °C during summer (July). On an average, the annual rainfall varies from 2000 mm to 3500 mm; the bulk of which is received during premonsoon and monsoon period (May to September). The quantum of precipitation is very low during winter. The relative humidity of the area varies from 55 to 90%.

The population density of the district is 832/km^2^ with a total population of 2,819,086 [45]. Majority of them are rural (89.70%) including indigenous people with varied socioeconomic conditions. The proportion of landless laborers is very large with 15.18% males and 4.31% females [45, 46]. The communities have their own distinct culture and belief system. The economy of the district is agriculture based, and rice is the major crop with a significant production of jute and other crops. The major occupation of the rural communities is farming. Their subsistence activity in farming includes rearing animals and growing paddy, jute, potato, other green vegetables, and maize in their marginal land holding. The rural population of the district also depends heavily on home gardens for their daily needs especially for day-to-day ailments and dietary and health supplements [10]. Therefore, every household has a home garden around their home with medicinal plants, vegetable, fruit, nuts, timber, and other plants.

### Ethnobotanical data collection

A multidisciplinary approach like collection of plant specimen, interview with structured questionnaire, and focus group discussion (FGD) was conducted for data collection following [47]. The selection of the study sites in the district, i.e., Cooch Behar peri-urban locality, Pundibari, Mathabhanga, Tufanganj I, and Balarampur, was purposive while home gardens were selected randomly. Home gardens are common features of Cooch Behar district due to its humid tropical location, and every household in the district has a home garden with sociocultural and livelihood significance [1, 7–9, 48, 49]. Therefore, Cooch Behar district with its five main locations was selected purposefully. A representative sample of 30 home gardens each from the five villages was selected randomly with a total of 150 home gardens [50, 51].

The data were collected from May 2017 to May 2018 through personal interviews using a pretested semi-structured questionnaire containing three sections related to socioeconomic, medicinal plant diversity in home gardens and their use, and therapy [27, 50–52]. Prior to the interview, we tried to establish rapport with the households and pretested the questionnaire for elimination, addition, and alteration with non-sampled households. The society of the study area is patriarchal with husband/father as the head of the household. We generally interviewed the head of the household. However, sometimes the wife, the eldest son, or the daughter of the household was also interviewed when the head of the household was not available [52]. The questions were asked in local language, but the responses were recorded in English. We also conducted ten FGDs, two in each selected area with 20–25 participants which included senior citizens, home garden owners, and local medicine man. Information from these discussions was helpful to interpret our result as this supplemented our household surveys.

### Sampling and data analysis

#### Data analysis

Considering the main objective of our study, we emphasized on documentation of the ethnomedicinal plants from the home gardens. However, floristic structure and composition of these plants were estimated considering the total species and population recorded from the home gardens. The coordinates of each home gardens were recorded by using GPS (Garmin Montana 680). The assessment of medicinal plant diversity and their composition in the home gardens was done by laying down a 10 × 10 m quadrat depending upon the area [33]. For the present study, only medicinally important species were taken into consideration based on the consumption and knowledge of the household members. Species identification was not a problem because most of the species grown were need-based and easily identified by the respondent. The plant specimens were collected, systematically tagged, pressed, dried, and mounted on herbarium sheets and were kept under presser for 2 weeks at room temperature. The specimens were poisoned in a solution of mercuric chloride and absolute alcohol (2 g mercuric chloride, dissolved in 1000 ml absolute alcohol) and mounted on the standard-sized herbarium sheets (11.5 × 17.5 in.^2^). The data noted in the field notebook during field survey was transferred to the label and pasted on the respective herbarium sheet on the right side at the bottom. The prepared specimens were then cross-checked and identified by a taxonomist of Angiosperms and Biosystematics Research Laboratory, Department of Botany, University of North Bengal and Department of Forestry, Uttar Banga Krishi Viswavidyalaya, West Bengal, India, with proper voucher numbers. For each home garden, the numbers of individuals for each medicinal species were recorded to determine frequency and important value index [53] to get the idea about the distribution and importance of species in these home gardens. Importance value index, a statistical quantity which gives an overall picture of a species and indicates their importance in the plant community, is the sum of relative frequency, relative density, and relative abundance. The frequency was determined by the formula:

$$ F=\frac{\mathrm{No}.\mathrm{of}\ \mathrm{home}\ \mathrm{gardens}\ \mathrm{in}\ \mathrm{which}\ \mathrm{a}\ \mathrm{species}\ \mathrm{occurs}}{\mathrm{Total}\ \mathrm{no}.\mathrm{of}\ \mathrm{home}\ \mathrm{gardens}}\times 100 $$

Use value (UV_i_) of the species was determined using the following formula according to [54],

$$ \mathrm{UVi}=\kern0.5em \frac{\sum Ui}{Ni} $$

where *U* is the number of times a species is cited and *N* is the number of informants. The use value of each species is therefore based objectively on the importance attributed by the informants and does not depend on the opinion of the researcher. The fidelity level (%) (FL) is measured according to [55] using the formula:

$$ \mathrm{FL}\%=\frac{I_{\mathrm{p}}}{I_u}\times 100 $$

where *I*_p_ is the number of informants who gave information of a given species as being important, while *I*_u_ is the total number of all informants mentioning important medicinal plants

To test the homogeneity of ethnomedicinal knowledge about the medicinal plants, the informant consensus factor (*F*_ic_) was used [56]. The *F*_ic_ for each of the recorded plant species was calculated using the following formula:

$$ {F}_{\mathrm{ic}}=\frac{N_{\mathrm{ur}}-{N}_{\mathrm{t}}}{N_{\mathrm{ur}}-1} $$

where *N*_ur_ is the number of use reports for a particular health problem and *N*_t_ is the number of species used for a particular health problem by all the informants.

To assess the importance of each species, cultural importance index (CI) was calculated by dividing the number of UR in use-category by the number of informants [57] using the following formula:

$$ {\mathrm{CI}}_{\mathrm{s}}=\sum \limits_{u={u}_1}^{u_{\mathrm{N}\mathrm{C}}}\sum \limits_{i={i}_1}^{i_{\mathrm{N}}}\frac{{\mathrm{UR}}_{\mathrm{ui}}}{N} $$

where UR is the number of use reports in various health problems (NC) and *N* is the total number of informants.

## Result and discussion

### Socioeconomic status

**Fig. 2:**
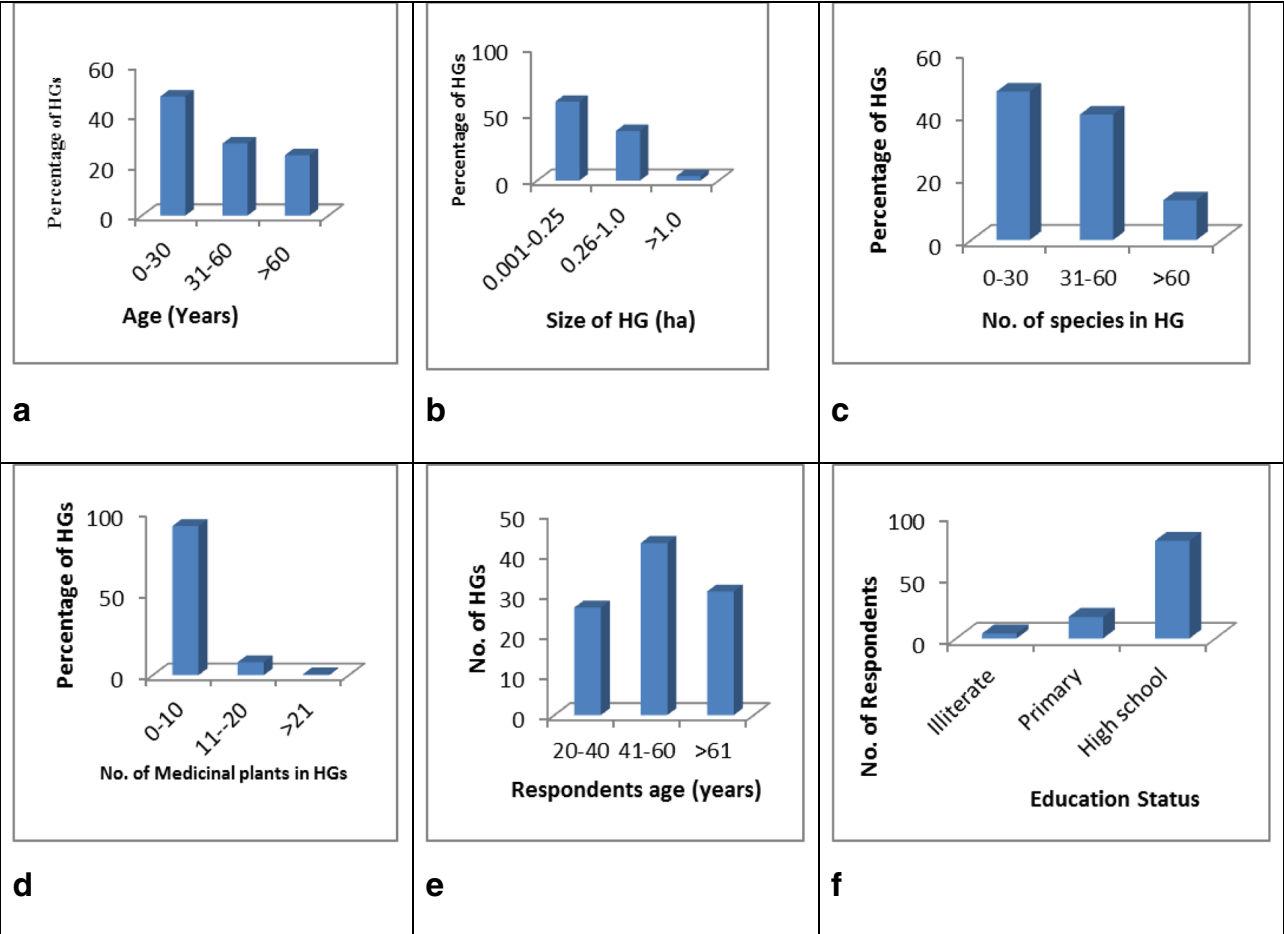
Basic information on home gardens collated from the study area. **a** Range of age of the home garden (HG) in years. **b** Range of size of the homestead garden in hectares. **c** Range of number of species. **d** Range of number of medicinal plants. **e** Age of the respondents in years. **f** Educational status of the home garden owners

All of the 150 surveyed home gardens were having ethnomedicinal plant species ranging from a minimum of 1 to a maximum of 21 species. Among these gardens, 46.66% of the families were of general caste followed by schedule caste (33.33), other backward class (18%), and scheduled tribes (2%). Almost half of the respondents (47.33%) were having education of higher secondary level followed by high school (31%) and least (11%) as illiterate. Majority of the respondents (90.66%) were male, and only 9.33% were female. Most of the respondents (42.66%) were of the age group of 41–60 followed by 30.66% of above 61 and 26.66% for 20–40 year age group. Most of the home garden owners (80%) were having dependency on home gardens for their different resources like ethnomedicine, food-based items, and other ecosystem services (Fig. 2a–f)

### Floristic composition

In the present study, a total of 260 plant species were recorded. Out of them, only 53 were utilized for different ethnobotanical applications (Additional file 1: Table S1). These 53 species belong to 35 families and 45 genera (Table 1). Home gardens had a specific relevance for conservation purposes due to their capacity to represent agro-biodiversity at multiple levels over small spaces and done socially [12, 58, 59]. A total of 281 plant species with 50 medicinal species from home gardens in Tehuacan Valley, Mexico, were reported [60] supporting the pattern of the present study. Documentation of 289 species of plants with 12% medicinal plants from 106 suburban home gardens in the Thimbirigaskatuwa suburban area, Western Sri Lanka, also supports the results of the present study [61]. Ethnomedicinal richness of 47 species has also been reported from home gardens of Pachmarhi Biosphere Reserve, India [62]. The reported results of [61, 62] also support to the contribution of ethnomedicinal plant species with total plant diversity from the home gardens. The earlier report of only 13 species of medicinal plants from the home gardens of North Bengal is very less as compared to the 53 species in the present study [63]. The variation may be due to the fact that the study conducted by [63] was restricted to 40 home gardens from the district of Cooch Behar. High diversity of species with an immediate use in the home is the most prominent feature of home gardens [64].

**Table 1:** Scientific name of documented species, families, ecological status, and other indices

Botanical name/accession number	Family	Local name	NI	F (%)	IVI	UV	FL (%)	CI
*Andrographis paniculata* (Burm. f.) Wall. (10061)^a^	Acanthaceae	Kalo meg/kal meg	16	10.67	2.66	0.093	93.75	0.048
*Hygrophila schulli* (Ham.) M.R. and S.M. Almeida Almeida (10068)^a^	Acanthaceae	Kulekhara	49	32.67	4.88	0.073	22.45	0.033
*Justicia adhatoda* L. (UBKV FOR 328)^b^	Acanthaceae	Basakpata	40	33.33	4.31	0.140	42.00	0.111
*Justicia gendarussa* Burm.f. (UBKV FOR 329)^b^	Acanthaceae	Bishalyokoroni	72	29.33	6.43	0.107	36.36	0.071
*Acorus calamus* L. (UBKV FOR 252)^b^	Acoraceae	Bogh	34	22.67	3.87	0.027	11.76	0.024
*Lannea coromandelica* (Houtt.) Merr.	Anarcardiaceae	Jiga	87	58.00	7.43	0.013	4.60	0.056
*Centella asiatica* (L.) Urban (10097)^a^	Apiaceae	Thankuni	89	59.33	7.56	0.467	78.65	0.091
*Alstonia scholaris* (L.) R. Br. (10089)^a^	Apocynaceae	Chatim	44	22.67	4.65	0.033	23.53	0.167
*Rauvolfia serpentinea* (L.) Benth. ex Kurz. (UBKV FOR 271)^b^	Apocynaceae	Sarpaganda	72	29.33	6.43	0.040	13.64	0.143
*Cocus nucifera* L. (UBKV FOR 309)^b^	Arecaceae	Narkel	160	51.33	11.06	0.067	12.99	0.091
*Asparagus racemosus* Willd. (UBKV FOR 255)^b^	Asparagaceae	Shatamuli	19	11.33	2.98	0.020	25.00	0.029
*Ageratum conyzoides* (L.) L. (UBKV FOR 29)^b^	Asteraceae	Gandhe	17	10.67	2.79	0.080	12.50	0.063
*Oroxylum indicum* (L.) Kurz (10111)^a^	Bignoniaceae	Surimala	29	15.33	3.73	0.080	52.17	0.037
*Ananas comosus* (L) Merr. (UBKV FOR 282)^b^	Bromeliaceae	Anaras	109	46.67	8.39	0.020	4.29	0.077
*Carica papaya* L. (UBKV FOR 298)^b^	Caricaceae	Paypay	88	36.00	7.27	0.027	7.41	0.200
*Terminalia arjuna* (Roxb. ex Dc.) Wight and Arn.(10,087)^a^	Combretaceae	Arjun	21	8.67	3.69	0.073	84.62	0.031
*Terminalia bellirica* (Gaertn.) Roxb. (UBKV FOR 45)^b^	Combretaceae	Bahera	7	4.67	2.06	0.047	22.22	0.036
*Terminalia chebula* Retz. (10124)^a^	Combretaceae	Haritaki	25	16.00	3.30	0.107	66.67	0.063
*Cuscuta europaea* L. (10121)^a^	Convolvulaceae	Sarnolata	28	18.67	3.47	0.040	46.43	0.077
*Bryophyllum pinnatum* (Lam.) Oken (10091)^a^	Crassulaceae	Patharkuchi	74	46.67	6.50	0.280	45.71	0.200
*Equisetum arvense* L. (10159)^a^	Equisetaceae	Harvanga	17	7.33	3.38	0.033	18.18	0.031
*Phyllanthus emblica* L. (UBKV FOR 263)^b^	Euphorbiaceae	Amloki	50	21.33	5.19	0.033	86.67	0.125
*Jatropha curcas* L. (UBKV FOR 264)^b^	Euphorbiaceae	Bharenda	44	24.00	4.61	0.080	33.33	0.037
*Euphorbia hirta* L. (10079)^a^	Euphorbiaceae	Lal dudhi	71	47.33	6.35	0.020	5.63	0.167
*Clerodendrum infortunatum* L. (10070)^a^	Lamiaceae	Vati	85	56.00	7.28	0.107	19.05	0.048
*Mentha arvensis* L.	Lamiaceae	Pudina	76	50.67	6.69	0.113	22.37	0.056
*Ocimum gratissimum* L.	Lamiaceae	Ram tulsi	19	12.67	2.86	0.027	21.05	0.031
*Ocimum sanctum* L. (UBKV FOR 267)^b^	Lamiaceae	Tulsi	135	90.00	10.65	0.533	89.63	0.077
*Leucas aspera* (Willd.) Link. (UBKV FOR 331)^b^	Lamiaceae	Kanchisa	101	67.33	8.37	0.067	6.93	0.333
*Vitex negundo* L*.* (10069)^a^	Lamiaceae	Nishinda	29	18.67	3.55	0.040	21.43	0.047
*Cinnamomum tamala* (Buch.-Ham.) T.Nees and Eberm.	Lauraceae	Tezpata	31	19.33	3.71	0.020	10.34	0.077
*Cinnamomum verum* J.Presl	Lauraceae	Dalchini	17	4.67	4.64	0.013	28.57	0.024
*Punica granatum* L. (UBKV FOR 345)^b^	Lythraceae	Dhalim	43	20.67	4.66	0.020	9.68	0.130
*Abroma augusta* (L.) L.f. (10104)^a^	Malvaceae	Allot kamal/ulatkambal	72	29.33	6.43	0.107	36.36	0.116
*Hibiscus rosa-sinensis* L. (UBKV FOR 324)^b^	Malvaceae	Jawa phul	143	80.67	10.68	0.027	33.16	0.029
*Azadirachta indica* A.Juss (UBKV FOR 256)^b^	Meliaceae	Neem	31	16.00	3.88	0.147	87.50	0.063
*Tinospora sinensis* (Lour.) Merr. (10137)^a^	Menispermaceae	Gulancha	14	9.33	2.53	0.040	14.29	0.167
*Mimosa pudica* L. (UBKV FOR 93)^b^	Mimosaceae	Lajjawati	58	38.67	5.48	0.093	5.17	0.094
*Streblus asper* Lour. (10084)^a^	Moraceae	Shaora	61	24.67	5.85	0.053	2.70	0.059
*Moringa oleifera* Lam. (UBKV FOR 336)^b^	Moringaceae	Sajana	23	12.00	3.39	0.053	16.67	0.067
*Averrhoa carambola* L. (10056)^a^	Oxalidaceae	Kamranga	24	14.67	3.27	0.120	9.09	0.043
*Piper betle* L. (10120)^a^	Piperaceae	Pan	128	85.33	10.18	0.280	32.81	0.167
*Piper nigrum* L. (UBKV FOR 268)^b^	Piperaceae	Golmarich	96	64.00	8.03	0.067	10.42	0.071
*Cynodon dactylon* (L.) Pers. (UBKV FOR 261)^b^	Poaceae	Dubbaghass	136	87.33	10.60	0.240	26.09	0.070
*Ziziphus mauritiana* Lam. (10122)^a^	Rhamnaceae	Kul	79	52.67	6.89	0.020	43.04	0.333
*Aegle marmelos* (L.) Correa (10062)^a^	Rutaceae	Bael	40	20.00	4.44	0.093	46.67	0.154
*Murraya koenigii* (L.) Spreng. (10063)^a^	Rutaceae	Curry pata	85	51.33	7.17	0.020	3.90	0.095
*Bacopa monnieri* (L.) Wettest	Scrophulariaceae	Brahmi	32	21.33	3.73	0.033	15.63	0.095
*Datura metel* L. (UBKV FOR 262)^b^	Solanaceae	Dhatura	52	34.67	5.08	0.153	44.23	0.108
*Aloe vera* (L.) Burm. f. (UBKV FOR 279)^b^	Xanthorrhoeaceae	Aloe vera	77	50.00	6.73	0.207	70.67	0.167
*Curcuma aeruginosa* Roxb.	Zingiberaceae	Jhanglihalud	28	18.67	3.47	0.033	14.29	0.273
*Curcuma longa* L. (10081)^a^	Zingiberaceae	Halud	108	72.00	8.84	0.247	34.26	0.077
*Zingiber officinale* Roscoe. (10082)^a^	Zingiberaceae	Ada	111	74.00	9.04	0.187	25.23	0.333

*NI* number of individuals, *F* frequency, *IVI* importance value index, *UV* use value, *FL* fidelity, *CI* cultural importance

^a^Species identified from Taxonomy of Angiosperms and Biosystematics Research Laboratory, Department of Botany, University of North Bengal

^b^Species identified from herbarium of Department of Forestry, UBKV

The contribution to 53 species was dominated by woody perennials (37.73%) followed by herb (28.30%), shrub 12 (22.64%), and climber 6 (11.32%), respectively (Table 2). The predominance of woody trees as a common rule in home gardens in the present study is well supported by the results of [65–68]. The contribution of fruit species is crucial for the diet of household members in terms of vitamins and fibers [69–72]. The dominance of woody trees in the present study is in contrast with the findings of [73] who found low numbers of trees in each garden, mostly because of their greater demand for space in Central Italy.

**Table 2:** Ethnomedicinal plant species with their uses and application procedure

Botanical name	Plant form	Used for	Parts used	Procedure	Reported cases
*Abroma augusta* (L.) L. f.	Shrub	Urine problem	New buds, leaf, and root	Juice of fresh leaves along with honey is given orally as expectorant. Juice of leaf is inhaled for bleeding nose (sinusitis). Dried powder of entire plant parts is given for bronchitis and cough.	Night wetting, jaundice, stomach disorder [23], blood dysentery, diarrhea [83], menstrual disorder [100, 101]
*Acorus calamus* L.	Herb	Fever and stomach disorders	Leaf, root	Juice of root is given orally in stomach disorders, bronchitis, and fever, and its small piece is chewed to clear the throat.	Joint pain, evil spirit [27], for removing animal lice [102]
*Aegle marmelos* (L.) Correa	Tree	Stomach problem	Fruit	Fruit and seed is used to treat stomach problem.	Stomach disorder, appetizer, dysentery [27, 103]
*Ageratum conyzoides* (L.) L.	Climber	Wounds	Leaf	Leaf juice is given to cure bleeding from cuts and wounds. Plant paste is applied to cure muddy wounds between toes during rainy season.	Cuts and wounds [27, 103]
*Aloe vera* (L.) Correa	Herb	Skin problem	Leaves	Leaf pulp is applied on skin burns and other problems.	For burnt skin, stomach disorder, body pain, diabeties, general health issues, cold, and cough [27, 81, 103]
*Alstonia scholaris* (L.) R. Br.	Tree	Cure skin problems	Bark	Bark paste is smeared of the infected part of the body.	Cold and cough, against stomach worms, lactation, snake bite, for ulcers, sores, and tumors [26, 27, 90, 103, 104]
*Ananas comosus* (L) Kurz	Herb	Kill stomach worms	Leaf	Leaf extract is used to kill worms.	For stomach worms, against scurvy [24, 26, 27, 103]
*Andrographis paniculata* (Brum.f.) Wall. ex Nees	Herb	Liver	Whole plant	Whole plant or leaf juice extract is used to cure liver problems.	Diabetes, liver problem, fever, cough and cold, stomach pain, Malaria [27, 90, 103, 104]
*Asparagus racemosus* Willd.	Herb	Diabetes and dysentery	Tuber	Fleshy root is dried, and its powder is consumed with water to treat diabetes and dysentery	Cuts and wounds, urine disorder, swelling, diabetes, dysentery, stomach disorders, improving memory [22, 27, 100, 101, 103]
*Averrhoa carambola* L.	Tree	Jaundice and dysentery	Fruit	Fresh fruit is eaten to treat jaundice, and dry fruit is used to treat dysentery.	Jaundice and liver problem [27, 103]
*Azadirachta indica* A. Juss	Tree	Fever and skin rashes	Leaf	Leaf sap is used to treat fever and acidity, and leaf is boiled with water and used as a bath water to treat skin rashes.	Allergy, pneumonia, appetite, stomach disorder, skin disorder, intestinal worms, eye problems, malarial fever, blood purification [26, 27, 100, 103, 105]
*Bacopa monnieri* (L.) Wettest	Herb	Indigestion	Whole plant	Boiled tender leaf is used for indigestion.	
*Bryophyllum pinnatum* (Lam.) Oken	Herb	Stomach stone	Leaf	Leaves in soaked water are used and drunk raw to treat stomach stone.	Cuts and wounds, for burnt skin, for gall bladder stone, piles, stomach problems [27, 84, 103]
*Carica papaya* L.	Tree	Jaundice	Fruit	Ripen fruit is consumed to treat against jaundice	Gastroenteritis, appetizer, digestion, cough, dysentery [23, 24, 27, 100, 101, 103]
*Centella asiatica* (L.) Urb.	Herb	Diarrhea/dysentery	Leaf	Leaf paste juice is taken orally for diarrhea/dysentery.	Jaundice, typhoid, dysentery, pain, constipation, indigestion, dog bite, appetizer [22, 27, 100, 101, 103]
*Cinnamomum tamala* (Buch.-Ham) T. Nees & Eberm.	Tree	Hypertension and diarrhea	Leaf and bark	Leaf and bark are consumed.	
*Cinnamomum verum* J.Presl	Tree	Gastrointestinal problems and diabetes	Bark and leaves	Raw small piece of bark is chewed to treat gastrointestinal problems and diabetes.	
*Clerodendrum infortunatum* L.	Herb	Stomach worm	Young buds	Paste of young shoot is taken orally.	
*Cocus nucifera* L.	Tree	Stomach problems and weight loss	Fruit water	Water present in coconut fruit is used in curing stomach problem and weight loss.	Weight loss, hair vitalizer, chicken pox scar [27, 76, 103]
*Curcuma aeruginosa* Roxb.	Herb	Inflammation	Rhizome	Fresh rhizome paste is used against inflammation.	
*Curcuma longa* L.	Herb	Antiseptic	Rhizome	Rhizome paste is applied as antiseptic. Rhizome and salt are taken to treat cough and cold.	Cuts and wounds, stomach disorder, lactation of animals, cough and cold, fever, bone fracture, blood purifier, snake bite [22, 27, 100, 103]
*Cuscuta europaea* L.	Climber	Jaundice or liver problem	Whole plant	Whole plant juice is used to treat jaundice.	Jaundice [27, 103]
*Cynodon dactylon* (L.) Pers.	Herb	Cut/wounds	Leaf	Young crush leaf paste is used to apply on cut wounds	Cuts and wounds, bleeding, vomiting, indigestion, piles, asthma [22–24, 27, 100, 101, 103]
*Datura metel* L.	Herb	Pain reliever	Leaf, flower and fruit	Few dried or fresh seeds are taken as pain reliever.	Cuts and wounds, diarrhea, tooth pain, asthmatic, cold and cough, anti-rabies [22, 37, 100, 101, 103]
*Equisetum arvense* L.	Herb	Bone disease	Whole plant	Paste of leaf and root are applied on fracture directly to reduce bone disease.	
*Euphorbia hirta* L.	Herb	Piles	Whole plant	Tender plant parts are crushed and consumed daily with milk to get relief against piles.	
*Hibiscus rosa-sinensis* L.	Shrub	Burning sensation, fatigue, skin diseases, and blood dysentery	Root, flower buds	Root extract is used to treat cough and fever. Flower bud is used to treat blood dysentery. Leaf paste is used to treat burning sensation, fatigue, and skin diseases.	Tonsillitis, dandruff, hair loss, burning sensation, fatigue, fever [25, 27, 103]
*Hygrophila schulli* (Hamilt.) M.R. & S.M. Almeida	Herb	Anemia	Leaf, tender stem	Leaves and young stems are boiled and taken daily to cure anemia.	
*Jatropha curcas* L.	Shrub	Tooth ache	Branch	Young branch is used as tooth brush to reduce pain.	Diarrhea, headache, cuts and wounds, digestion, gum problems [22, 27, 83, 84, 103]
*Justicia Adhatoda* L.	Herb	Bronchitis, cold, and cough	Leaf	Leaf juice is used to treat chronic bronchitis, cough, and cold.	Cough and cold, paralysis, allergy, cuts and wounds, piles, leprosy and diabetes, chronic bronchitis [25, 27, 86, 103]
*Justicia gendarussa* Burm.f.	Herb	Headache, cut, and wounds	Leaf	Crushed leaves are placed on cuts with the extract for healing. It is applied on forehead for treating headache.	Cancer, antiseptic, headache, cuts and wounds [27, 103]
*Lannea coromandelica* (Houtt.) Merr.	Tree	Piles and wounds	Gum	Gum is applied directly on piles and wounds.	
*Leucas aspera* (Willd.) Link.	Herb	Stomach disorder, swelling, and stop bleeding	Leaf	Leaf juice is used to cure stomach disorder; hand-crushed leaves are inhaled to cure headache; leaf paste is applied to stop cut bleeding.	Stomach disorder, swelling, headache, body pain, cuts and wounds, tooth pain, bleeding [27, 103]
*Mentha arvensis* L.	Herb	Flatulence, diarrhea, and nausea	Leaves	Bruised leaves are applied to get relief from headache, and leaf extract juice is administered orally against vomiting, nausea, flatulence, and diarrhea.	
*Mimosa pudica* L.	Herb	Orchitis and depurative	Leaf and bark	Decotation of leaf and bark is used to control orchitis and used as depurative.	Eye problem, orchitis and depurative, infertility, tooth ache [22, 27, 83, 103]
*Moringa oleifera* Lam.	Tree	Blood pressure and gastroenteritis	Leaf and fruits	Tender leaf juice and cooked fruits are taken to balance blood pressure.	Blood pressure, gastroenteritis, cold and cough, body pain, cut and wound, leucoderma, liver disorder, urine problems [27, 100, 101, 104]
*Murraya koenigii* (L.) Spreng.	Shrub	Fever and diarrhea	Leaf, bark, and root	Leaf juice is consumed to control black fever and diarrhea.	Gastroenteristis, black fever and diarrhea, diabetes mellitus [22, 27, 83, 103, 104]
*Ocimum gratissimum* L.	Herb	Fever, cough and cold, headache, nausea, diarrhea, dysentery, and skin diseases	Leaves, flower	Decoction of plant is given to treat fever, cough, cold, headache, nausea, diarrhea, dysentery, and skin diseases.	
*Ocimum sanctum* L.	Herb	Cold and cough	Leaf	Leaves are used to treat cough and cold.	Cough and cold, neck pain, cancer, gastroenteritis, asthma, urinary disorder [22, 25, 27, 103]
*Oroxylum indicum* (L.) Kurz	Tree	Stomach pain	Fruits, seed, and bark	Paste of hydrated fruit or seed or bark is applied for stomach pain and chest pain and is used as appetizer and against jaundice.	Jaundice, cuts and wounds, body pain, liver problems, stomach pain, chest pain, appetizer, asthma [22, 25–27, 84, 103, 104]
*Phyllanthus emblica* L.	Tree	Stomach problem/gastritis	Fruit	Dry paste or fruits are used to chew or eat orally for stomach relief.	Hair loss, stomach pain, liver problem [23, 26, 27, 103]
*Piper betle* L.	Climber	Gastrointestinal problems, cold, and cough	Leaves and root	Fresh leaf is chewed with beetle nut to cure gastrointestinal problems, and dry root is chewed whole day to cure throat-related problems.	
*Piper nigrum* L.	Climber	Asthmatic problems, cold, cough and rheumatism	Seed	Seeds are boiled with sugar and salt and consumed three to four times a day to control asthmatic problems, cold, cough, and rheumatism.	Cough and cold, asthma, constipation, indigestion, throat infection
*Punica granatum* L.	Tree	Increase blood	Fruit	Fresh fruits are eaten to increase blood.	Nose bleeding, diarrhea, fever, indigestion [27, 100, 101, 103, 104]
*Rauvolfia serpentine* (L.) Benth. ex Kurz.	Herb	Fever	Root	Leaf juice is used as a remedy for the removal of opacities of cornea. Root paste is applied on cuts, wounds, or boils. Root infusion is given orally for intestinal disorders.	Paralysis, diabetes, fever, cuts and wounds, pneumonia, jaundice, stomach worm, dysentery, reliving hypertension and blood pressure, intestinal disorder [22, 25, 27, 84, 103, 104]
*Streblus asper* Lour.	Tree	Toothache	Bark, latex and root	Tender stem is used as toothbrush to cure toothache.	
*Terminalia arjuna* Roxb. ex DC) Wight & Arn.	Tree	Cardiac trouble	Bark	Bark decoction is taken in empty stomach to treat cardiac trouble.	Asthma, heart problem, diabetes, stomach disorder, appetizer, skin disease, leucoderma, indigestion, chest pain, tuberculosis, cardic trouble [22, 25, 27, 86, 103, 104]
*Terminalia bellirica* (Gaertn.) Roxb.	Tree	Dyspepsia (indigestion)	Fruit	Dried fruit is used to treat dyspepsia and as cooling agent.	Cough and cold, stomach disorder, indigestion, gastroenteritis, skin diseases, leucoderma, cooling agent, [13, 20, 22, 23, 27, 103]
*Terminalia chebula* Retz.	Tree	Stomach problem	Fruit	Fruit is used against stomach disorder.	Appetizer, cough and cold, gastroenteritis, jaundice, pneumonia, liver problems, indigestion, urinary disorder, tonsillitis, fever [23, 25, 27, 100, 102, 103, 105]
*Tinospora sinensis* (Lour.) Merr.	Climber	Rheumatism and jaundice	Bark	Bark is soaked overnight in water and is consumed in the morning against rheumatism and jaundice.	Stomach pain, diabetes, rheumatism and jaundice, urination, piles bleeding, appetite, diarrhea [22, 27, 100, 101, 103, 104]
*Vitex negundo* L.	Shrub	Whitening of hair	Leaf	Leaves prevent whitening of hair.	Fever, gout, diarrhea, cardic disorder, headache, bone fracture, body swelling [22, 27, 83, 90, 103]
*Zingiber officinale* Roscoe.	Herb	Digestive, stimulant, and cold and cough	Rhizome	Rhizome is used as digestive stimulant.	
*Ziziphus mauritiana* Lam.	Tree	Vomiting	Fruits and seed	Fruits and seeds are consumed with salt to control vomiting.	

Lamiaceae was the dominant family with five genera and six species followed by Acanthaceae with three genera and four species, Euphorbiaceae with three genera and three species, and Combretaceae with one genera and three species. The highest number of individuals (160) was reported for *Cocus nucifera* followed by *Hibiscus rosa-sinensis* (143) (Table 1)*. Ocimum sanctum* was the most frequent species followed by *Cynodon dactylon* (87.33%), *Piper betal* (85.33%), *Hibiscus rosa-sinensis* (80.67%), and *Zingiber officinale* (74.00%). The least frequent species recorded was *Terminalia bellirica*. The highest frequency (%) was observed in *Ocimum sanctum* because this species is commonly used for cold and cough and taken with hot water instead of tea everyday morning. The high individual occurrence of *Cocus nucifera* in these home agroforestry gardens is due to the economic viability and home consumption. The important value index (IVI) is used to determine the overall importance of each species in the community structure. The recorded average value of IVI was 5.68 with range of 2.05 to 11.06 (Table 1). The highest value of IVI (11.06) was recorded for *Cocus nucifera* and lowest (2.05) for *Terminalia bellrica.* The dominance of *Cocus nucifera* in home gardens of North Bengal was also reported by [1].

### Parts used and ailments treated

**Fig. 3:**
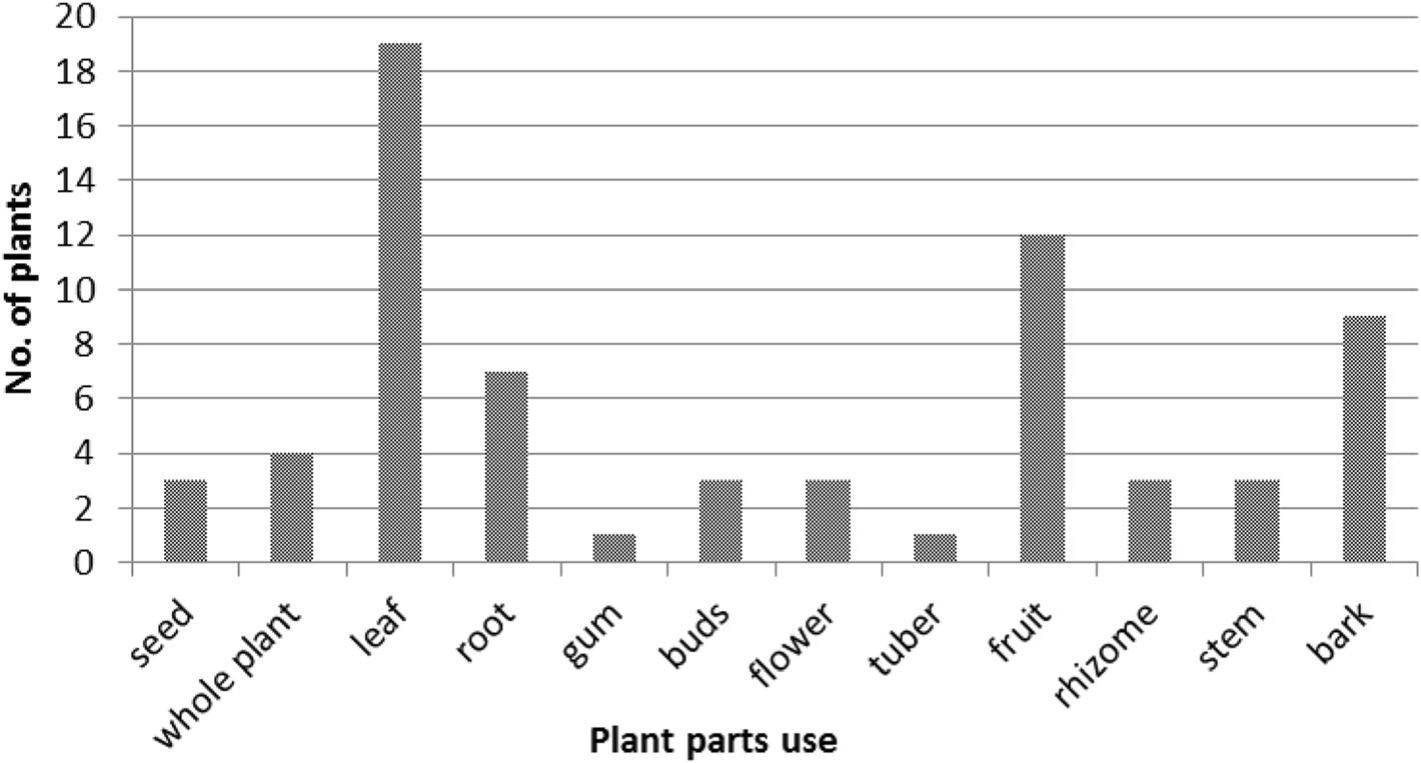
Contribution of different plant parts to ethnomedicine

**Fig. 4:**
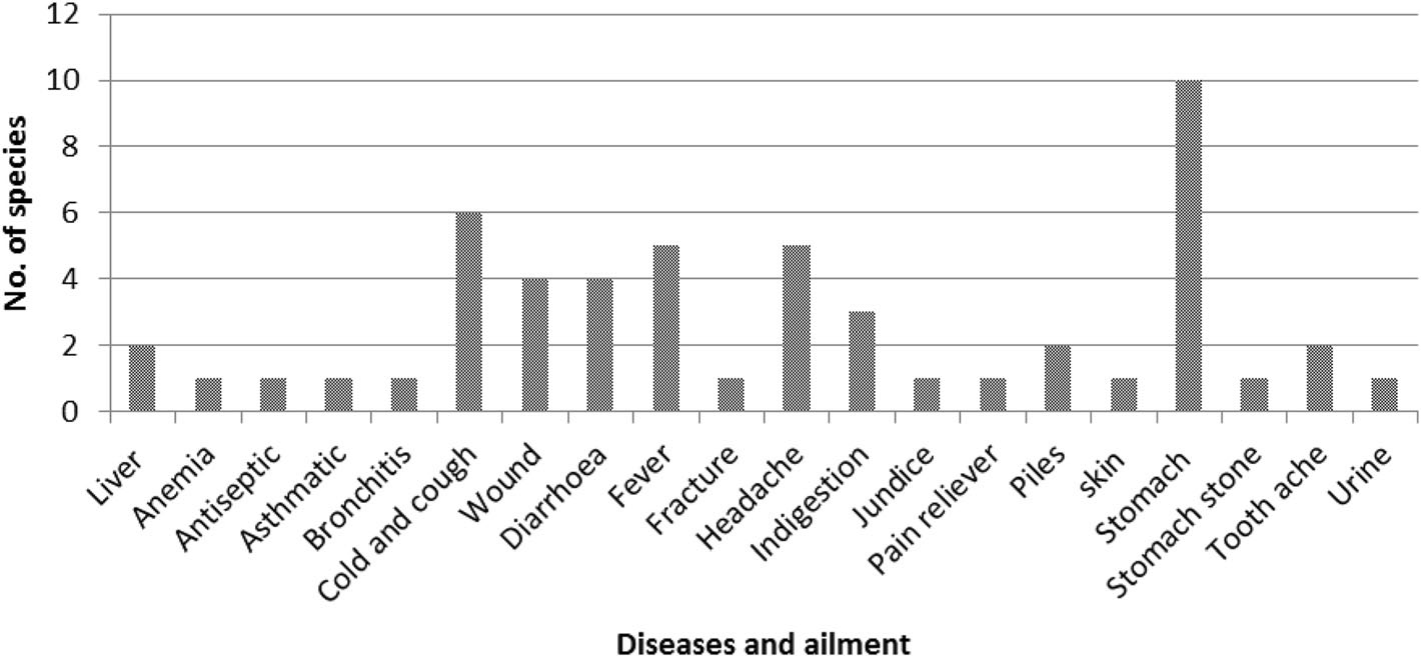
Number of species used to treat prevailing ailments in the area

The present study reported the leaf of 19 species as dominant plant part used as medicinal part followed by fruit (12), bark (9), root (7), and whole plant (4) (Fig. 3; Table 2). Leaves of the ethnomedicinal plants as dominant plant part to treat different diseases have also been reported by several studies [24, 25, 27, 73]. Fruit was also reported as dominant and widely used part for traditional medicines [23, 74]. These can further lead to a scientific assessment of the traditional medicines used, which may provide a lead in drug development [75, 76]. Botanically derived medicinal plants played a major role in human societies throughout history and prehistory [19, 20]. Traditional medicinal use of plants is strongly related to physiological and pharmacological activity of active plant ingredients [67]. The plant parts utilized were either taken in the form of juice, paste, decoction, powder, infusion, and chewing raw plant parts. These utilized plant parts are used to cure 20 ailments prevailing among the dependent households (Table 2). The maximum species (10) were documented for the treatment of stomach disorders followed by cough and cold (6), wounds, and jaundice of four species (Fig. 4; Table 2).

The stomach disease is prevailing in the region among all age groups due to iron rich water. The use of *Aegle marmelos* in stomach disorder in the present study has been supported by the findings of [77, 78] who reported it for digestive disorders. *Azadirachta indica* used in fever in the present study is also reported for the same by [79, 80] among different dependent communities. The use of plants as food and medicine is common among Bengali women in Sylhet, Bangladesh [81]. On the regional scale, the maximum number of medicinal plants has been reported from Uttarakhand [82] followed by Sikkim and North Bengal [83]. The hill indigenous and aborigines of West Rarrh region of West Bengal use 46 plant species ethnomedicinally in the form of infusion, decoction, oil paste, and latex either as a sole drug or in combination to treat various ailments. A study reported that 91 species of medicinal plants are used for the treatment of skin disease by the indigenous population of Darjeeling Himalayas [83]. The Rava tribe of Jalpaiguri district of West Bengal has been reported to use 41 plant species as ethnomedicine [84]. The most frequently utilized plant parts are bark, leaves, roots, branches, stem, fruits, and seeds [22, 85, 86]. Additionally, some of them have medicinal value in their flowers, rhizomes, tubers, and wood. In some cases, the whole plant including the roots was utilized [22, 30]. Most of the ethnobotanical studies confirmed that the leaves are the major portion of the plant used in the treatment of diseases [87–91]. We also compared these 53 medicinal plant species with the previously available literature and found 37 of them have also been reported earlier for their different uses from this region (Table 2).

### Use value, fidelity, informant consensus factor, and cultural importance index

The range of use value for the documented species varied from the highest of 0.53 to the lowest of 0.006 (Table 1). The highest value of 0.53 was recorded for *Ocimum sanctum* followed by *Centella asiatica* (0.46) and *Piper betle* (0.28), and least value of 0.013 was observed each for *Lennea coromandelica* and *Cinnamomum zeylanicum*. The high use value (0.53) recorded for *Ocimum sanctum* in the study area may be due to the medico-religious significance among Hindu communities as has been described in their religious books. The highest UV was 0.48 for the species *Zingiber officinale* as ethnomedicinal plant has also been reported by [92] from Kurdistan, Iraq, and is in the reported range of results of the present study. Similar values of use value, for example, 0.51, 0.53, and 0.59, on ethnomedicinal species have been reported by [93–95], respectively, from different regions of the globe and are in well support of the present study. The fidelity value (%) of the species ranged from 2.29 to 93.75%. The highest value of fidelity (93.75%) was observed in *Andrographis paniculata* followed by *Azadirachta indica* (87.5%) and *Ocimum sanctum* (89.6%). The lowest value of fidelity (2.70%) was observed in *Streblus asper* (Table 2). The high-value species is the important medicinal plant present in the home garden, and almost every informant was aware about its values. Studies of traditional agricultural systems in tropical regions of the world provide important information for understanding ecological processes associated with sustainable management of natural resources [96].

**Fig. 5:**
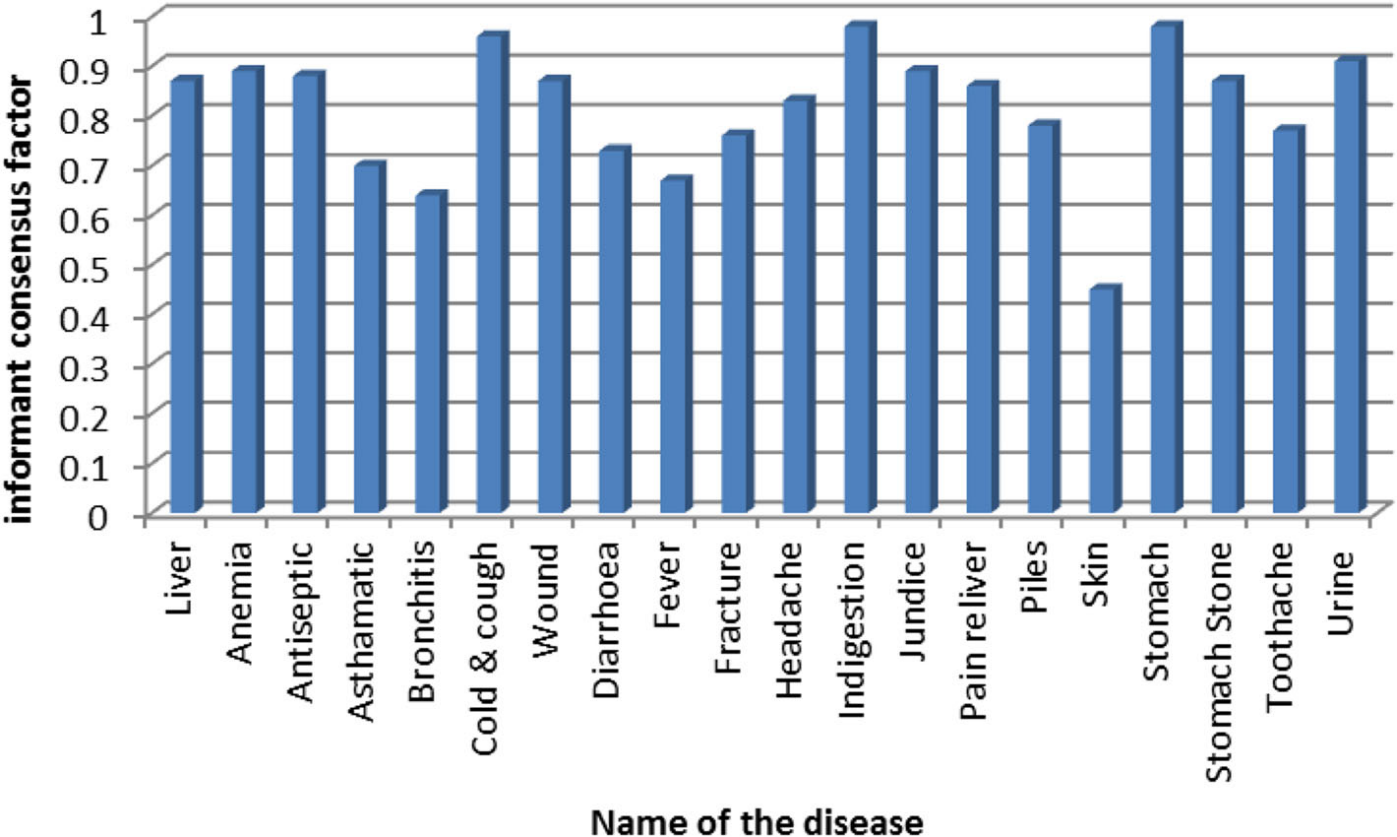
Values of informants consensus factor (*F*_ic_) for different disease category

The reported use of medicinal plants was categorized into 20 broad diseases (Fig. 4) to identify species with a particular importance in culture (Fig. 5). The highest value of *F*_ic_ was reported for indigestion and stomachache (0.98) followed by cold and cough (0.96). Lowest values of *F*_ic_ (0.45) were recorded for skin diseases followed by bronchitis (0.64) and fever (0.67). Also, the highest *F*_ic_ value of 0.96 has been reported for gastrointestinal diseases from Pakistan. Highest *F*_ic_ values of 0.80 for gastrointestinal and kidney problems followed by respiratory infections (0.72) and skin infections (0.73) have also been reported by other authors [98]. The study conducted by [24] has also reported the highest number of species for diseases like indigestion and stomachache from the forest fringe communities of North Bengal. These ethnomedicinal plants are important elements of healthcare in the region and are regarded as important cultural components. The highest value of cultural importance (CI; 0.45) was observed for *Abroma augusta* followed by 0.33 for *Rauvolfia serpentina*, *Streblus asper*, and *Vitex negundo*. The lowest value of CI (0.024) was observed for *Emblica officinalis* followed by 0.029 each for *Euphorbia hirta* and *Hygrophila chulli*. The cultural importance of medicinal plants has been reported by several authors throughout the globe [97–99] and hence can allow a resolution regarding the actual status of conservation of plants. The multiple uses of plant species for different diseases seem to be an important cause for the widespread adaptation of these medicinal plants. Ethnobotanical studies can further lead to a scientific assessment of the traditional medicines used which may provide a lead in drug development [75, 76]. A detailed knowledge of the pharmacological effect of herbal drugs is therefore necessary for effective therapy of diseases. One of the major concerns regarding the use of herbal medicines is, however, their safe usage.

## Conclusion

The reporting of 260 species of plant diversity and 53 species as medicinal plants from a cluster of a village is an important finding of diversity maintained by the home gardens. In addition, the documentation of ecological status and ethnomedicinal practices provided important knowledge for understanding the ecological processes and their sustainable management. The study clearly indicated that a wide range of local people is dependent on home gardens, and substantial number species with medicinal values are maintained in home gardens. These gardens act as conservation centers for many of these species. The results of the study confirm that household owners have good idea about the application of these medicinal plant parts. The application of ethnomedicine from the home garden also shows their role in maintaining the family healthcare system. These findings suggest that medicinal plants and folk medicines used by the communities in Cooch Behar district of West Bengal may be an initiation for further ethnomedicine research and could lead to the conservation of the medicinal flora. The woody trees in the garden apart from providing ethnomedicine also provide good opportunities for small-scale farmers to seize the carbon market opportunities in the future.

## Additional file

Additional file 1: **Table S1.** Details of collected species as supplementary file. (DOCX 24 kb)
