# Supplementary material for: Floristic composition and utilization of ethnomedicinal plant species in home gardens of the Eastern Himalaya
Source: J Ethnobiol Ethnomed. 2019 Feb 19;15:14. doi: 10.1186/s13002-019-0293-4 (PMC6380006; doi:10.1186/s13002-019-0293-4)
Supplement: Supplementary file 1 — Table S1. Details of collected species as supplementary file. (DOCX 24 kb) [file 13002_2019_293_MOESM1_ESM.docx]

| **Table S1: Details of collected species as supplementary file** | | |
| --- | --- | --- |
| **Family** | **Scientific Name** | **Collection no/A. no** |
| Acanthaceae | *Andrographis paniculata* (Burm. f.) Wall. | C101/10061 |
|  | *Barleria cristata* L. | C102/10067 |
|  | *Hygrophila schulli* (Ham.) M.R. & S.M. Almeida | C103/10068 |
|  | *Justicia gendarussa* Burm.f. | C104 |
|  | *Phlogacanthus thyrsiflorus* Nees. | C105 |
|  | *Justicia adhatoda* L. | C106/10133 |
| Acoraceae | *Acorus calamus* L. | C107/10047 |
| Amaranthaceae | *Achyranthes aspera* L. | C108/10047 |
|  | *Alternanthera brasiliana* (L.) Kuntze. | C109 |
|  | *Amaranthus spinosus* L. | C110/10051 |
|  | *Amaranthus tricolor* L. | C111 |
|  | *Amaranthus viridis* Hook. F. | C112 |
|  | *Celosia sp*. | C113/10048 |
|  | *Gomphrena globosa* L. | C114/10049 |
| Amaryllidaceae | *Allium sativum* L. | C115 |
| Anacardiaceae | *Lannea coromandelica* (Houtt.)Merr. | C116 |
|  | *Mangifera indica* L | C117 |
| Annonaceae | *Polyalthia longifolia* Sonn. Thwaites | C118/10100 |
| Apiaceae | *Centella asiatica* (L.) Urban | C119/10097 |
|  | *Coriandrum sativum* L. | C120 |
|  | *Daucus carota* L. | C121 |
| Apocynaceae | *Allamanda cathartica* L. | C122/10132 |
|  | *Alstonia scholaris* (L) R. Br. | C123/10089 |
|  | *Asclepias curassavica* L. | C124/10148 |
|  | *Carissa carandas* L. | C125 |
|  | *Catharanthus roseus* (L) G. Don | C126 |
|  | *Rauvolfia serpentina* (L.) Benth ex Kurz. | C127 |
|  | *Tabernaemontana divaricata* R.Br. ex Roem. & Schult. | C128 |
|  | *Thespesia populnea* (L.) Sol. ex Corrêa | C129 |
|  | *Wrightia antidysenterica* (L.) R. Br. | C130 |
| Araceae | *Alocasia macrorrhiza* (L.) G. Don | C131 |
|  | *Amorphophallus paeoniifolius* (Dennst.) Nicolson | C132 |
|  | *Anthurium andraeanum* Linden ex Andre | C133 |
|  | *Colocasia esculenta* (L.) Schott | C134 |
|  | *Epipremnum aureum* (Linden & Andre) G. S Bunting | C135 |
|  | *Typhonium trilobatum* (L.) Schott | C136/10110 |
| Araucariaceae | *Araucaria araucana* (Molina) k. Koch | C137 |
| Arecaceae | *Areca catechu* L. | C138 |
|  | *Borassus flabellifer* L. | C139 |
|  | *Calamus erectus* Roxb. | C140 |
|  | *Caryota urens* L. | C141 |
|  | *Cocus nucifera* L. | C142 |
|  | *Phoenix sylvestris* (L.) Roxb. | C143 |
| Aroideae | *Caladium* spp | C144 |
| Asparagaceae | *Asparagus racemosus* willd. | C145 |
| Asteraceae | *Ageratum conyzoides* (L.) L. | C146 |
|  | *Ageratum houstonianum* Mill. | C147 |
|  | *Artemisia vulgaris* L. | C148/10065 |
|  | *Centratherum punctatum* Cass. | C149/10131 |
|  | *Chrysanthemum indicum* L. | C150 |
|  | *Crassocephalum crepidioides* (Benth) S. Moore | C151/10064 |
|  | *Dahlia apiculata* (Sherff) P.D.Sørensen | C152 |
|  | *Eclipta prostrata* (L.) L. | C153/10134 |
|  | *Emilia sonchifolia* (L.) DC. ex DC. | C154 |
|  | *Gerbera ambigua* (Cass.) Sch.Bip. | C155 |
|  | *Mikania micrantha* Kunth | C156 |
|  | *Spilanthes calva* DC. | C157/10066 |
|  | *Parthenium hysterophorus* L. | C158/10125 |
|  | *Tagetes erecta* L. | C159 |
| Athyriaceae | *Diplazium esculentum* (Retz.) Sw. | C160/10128 |
|  | *Diplazium molokaiense* W.J.Rob | C161 |
| Basellaceae | *Basella alba* L. | C162 |
| Bignoniaceae | *Oroxylum indicum* (L.) Kurz | C163/10111 |
| Bixaceae | *Bixa orellana* L. | C164/10092 |
| Bombaceae | *Bombax ceiba* L. | C165 |
|  | *Ceiba pentandra* (L.) Geartn. | C166 |
| Boraginaceae | *Ehretia acuminata* R.Br. | C167 |
|  | *Heliotropium indicum* L. | C168 |
| Brassicaceae | *Brassica juncea* (L.) Czern. | C169 |
|  | *Brassica oleracea* L. | C170 |
|  | *Brassica rugosa* (Roxb.) Prain | C171 |
|  | *Raphanus caudatus* L. | C172 |
| Bromelioideae | *Ananas comosus* (L.) Merr. | C173 |
| Calophyllaceae | *Calophyllum inophyllum* L. | C174/10085 |
| Cannabaceae | *Cannabis sativa* L. | C175/10112 |
| Caricaceae | *Carica papaya* L. | C176 |
| Chenopodiaceae | *Chenopodium album* L. | C177 |
| Combretaceae | *Terminalia arjuna* (Roxb. ex Dc.) Wight & Arn. | C178/10087 |
|  | *Terminalia bellirica* (Gaertn.) Roxb. | C179 |
|  | *Terminalia chebula* Retz. | C180/10124 |
| Commelinaceae | *Commelina benghalensis* L. | C181/10101 |
| Convolvulaceae | *Cuscuta europaea* L. | C182/10121 |
|  | *Ipomoea aquatica* Forssk. | C183 |
| Costaceae | *Cheilocostus speciosus* (J.koenig) C.D. Specht | C184 |
| Crassulceae | *Bryophylium pinnatum* (Lam.) Oken | C185/10091 |
| Cucurbitaceae | *Benincasa hispida* (Thunb.) Cogn. | C186 |
|  | *Cucumis sativus* L. | C187 |
|  | *Cucurbita maxima* Duchesne | C188 |
|  | *Lagenaria siceraria* (Molina) Standl. | C189 |
|  | *Luffa aegyptiaca* Mill. | C190 |
|  | *Luffa cylindrica* (L.) M. Roem. | C191 |
|  | *Momordica charantia* L. | C192 |
|  | *Momordica dioica* Roxb. ex. Willd. | C193 |
|  | *Trichosanthes cucumerina* L. | C194 |
|  | *Trichosanthes dioica* Roxb. | C195 |
| Cyperaceae | *Cyperus rotundus* L. | C196/10077 |
| Dilleniaceae | *Dillenia indica* (L.) | C197 |
| Dioscoreaceae | *Dioscorea belophylla* (Prain) Voigt ex Haine | C198 |
| Dipterocarpaceae | *Shorea robusta* Gaertn. | C199 |
| Ebenaceae | *Diospyros malabarica* (Desr.) Kostel. | C200/10113 |
| Elaeocarpaceae | *Elaeocarpus floribundus* Blume | C201 |
|  | *Elaeocarpus serratus* L. | C202/10119 |
| Equisetaceae | *Equisetum arvense* L. | C203/10159 |
|  | *Hippochaete debilis* (Roxb. ex Vaucher) Ching. | C204/10098 |
| Eryngium | *Eryngium foetidum* L. | C205/10108 |
| Euphorbiaceae | *Codiaeum variegatum* (L.) Rumph. ex A.Juss. | C206 |
|  | *Codiaeum variegatum* var. pictum (L.) A.juss | C207 |
|  | *Phyllanthus emblica* L. | C208 |
|  | *Euphorbia hirta* L. | C209/10079 |
|  | *Euphorbia pulcherrima* Wild. ex Klotzsch | C210 |
|  | *Jatropha curcas* L. | C211 |
|  | *Mallotus tetracoccus* (Roxb.) Kurz | C212/10080 |
| Fabaceae | *Acacia auriculiformis* Benth. | C213/10055 |
|  | *Albizia odoratissima* (L. f.) Benth. | C214 |
|  | *Albizia procera* (Roxb.) Benth. | C215 |
|  | *Albizia saman* (Jacq.) Merr. | C216 |
|  | *Butea monosperma* (Lam.) Tuab | C217/10052 |
|  | *Cajanus cajan* (L.) Millsp. | C218 |
|  | *Cassia fistula* (L.) | C219/10059 |
|  | *Senna tora* (L.) Roxb. | C220 |
|  | *Pongamia pinnata* (L.) Pierre | C221/10054 |
|  | *Clitoria ternatea* L. | C222/10058 |
|  | *Crotalaria pallida* var. obovata (G.Don) Polhill | C223/10146 |
|  | *Delonix regia (*Hook.) Raf. | C224 |
|  | *Desmodium laxiflorum* DC. | C225/10060 |
|  | *Lablab purpureus* (L.) Sweet | C226 |
|  | *Erythrina variegata* L. | C227/10057 |
|  | *Leucaena leucocephala* (Lam.) de Wit | C228 |
|  | *Mimosa pudica* L. | C229 |
|  | *Pisum sativum* L. | C230 |
|  | *Saraca asoca* (Roxb.) Willd. | C231/10053 |
|  | *Sesbania grandiflora* (L.) Pers. | C232 |
|  | *Tamarindus indica* L | C233 |
| Heliconiaceae | *Heliconia abaloi* G.Morales | C234 |
| Hypoxidaceae | *Curculigo annamitica Gagnep.* | C235/10129 |
| Lamiaceae | *Clerodendrum infortunatum* L. | C236/10070 |
|  | *Cinnamomum verum* J.Presl | C237 |
|  | *Leucas aspera* (Willd.) Link | C238/10103 |
|  | *Ocimum gratissimum* L. | C239 |
|  | *Ocimum sanctum* L. | C240 |
|  | *Tectona grandis* L.F. | C241 |
|  | *Vitex negundo* L. | C242/10069 |
| Lauraceae | *Cinnamomum tamala* (Buch.-Ham.) T.Nees & Eberm. | C243 |
|  | *Machilus fasciculata* H.W. Li | C244/10090 |
| Lygodiaceae | *Lygodium japonicum* (Thunb.) Sw. | C245/10149 |
|  | *Lagerstroemia speciosa* L. Pers. | C246 |
|  | *Lawsonia inermis* L. | C247 |
|  | *Punica granatum* L. | C248 |
| Magnoliaceae | *Magnolia champaca* (L.) Baill. ex Pierre | C249 |
| Malvaceae | *Abelmoschus esculentus* (L) Moench | C250/10154 |
|  | *Abelmoschus angulosus* Wall. ex Wight & Arn. | C251 |
|  | *Abroma augusta* (L.) L.f. | C252/10104 |
|  | *Hibiscus rosa-sinensis* L. | C253 |
|  | *Malva verticillata* L. | C254 |
|  | *Malvaviscus arboreus* cav. | C255/10099 |
|  | *Sida acuta* Burm. f. | C256/10126 |
|  | *Sida rhombifolia* L. | C257/10144 |
|  | *Triumfetta rhomboidea* Jacq. | C258/10141 |
|  | *Mentha syvestris* Linn. | C259/10158 |
| Melastomataceae | *Melastoma malabathricum* L. | C260 |
| Meliaceae | *Amoora rohituka* (Roxb.) Wight & Arn. | C261 |
|  | *Amoora wallichii* King | C262 |
|  | *Aphanamixis polystachya* (Wall.) R. Parker | C263/10115 |
|  | *Azadirachta indica* A. Juss. | C26410086 |
|  | *Chukrasia tabularis* A. Juss. | C265/10114 |
|  | *Melia azedarach* L. | C266 |
|  | *Swietenia macrophylla* King. | C267 |
|  | *Toona australis* (F. Muell.) Harms | C268 |
| Menispermaceae | *Tinospora sinensis* (Lour.) Merr. | C269/10137 |
| Moraceae | *Artocarpus heterophyllus* Lam. | C270/10088 |
|  | *Artocarpus lacucha* Buch.-Ham. | C271 |
|  | *Ficus benghalensis* L. | C272 |
|  | *Ficus carica* L. | C273 |
|  | *Ficus elastica* Roxb. ex Hornem. | C274 |
|  | *Ficus hispida* L.f | C275 |
|  | *Ficus lacor* Buch.-Ham | C276 |
|  | *Ficus racemosa* L. | C277 |
|  | *Ficus religiosa* L. | C278/10095 |
|  | *Morus alba* L. | C279 |
|  | *Streblus asper* Lour. | C280/10084 |
| Moringaceae | *Moringa oleifera* Lam. | C281 |
| Mimosaceae | *Mimosa pudica* L. | C282 |
| Musaceae | *Musa* sp. | C283 |
| Myrtaceae | *Callistemon lanceolatus* (Sm.) Sweet | C284/10118 |
|  | *Psidium guajava* L. | C285 |
|  | *Syzygium aromaticum* (L.) Merr. & L.M. Perry | C286 |
|  | *Syzygium cumini* (L.) Skeels | C287 |
|  | *Syzygium jambos* (L.) Alston | C288 |
|  | *Syzygium samarangense* (Blume) Merr. & L.M. Perry | C289 |
| Nelumbonaceae | *Nelumbo nucifera* Gaertn. | C230 |
| Nyctaginaceae | *Bougainvillea spectabilis* Willd. | C291 |
| Oleaceae | *Nyctanthes arbor-tristis* L. | C292/10102 |
| Oxalidaceae | *Oxalis corniculata* L. | C293/10145 |
|  | *Oxalis debilis* var. corymbosa (DC.) Lourteig | C294 |
| Oxalidales | *Averrhoa carambola* L. | C295 |
| Papilinoideae | *Dalbergia sissoo* DC. | C296 |
| Phyllanthaceae | *Baccaurea ramiflora* Lour. | C297/10123 |
|  | *Bischofia javanica* Blume | C298 |
| Piperaceae | *Piper nigrum* L. | C299 |
|  | *Piper betle* L. | C300/10120 |
| Plantaginaceae | *Plantago ovata* Forssk | C301 |
|  | *Scoparia dulcis* L. | C302/10153 |
| Poaceae | *Axonopus compressus* (Sw.) P. Beauv. | C303/10142 |
|  | *Brachiaria ramosa* (L.) Stapf. | C304/10072 |
|  | *Brachiaria reptans* (L.) C.A.Gardner & C.E.Hubb. | C305 |
|  | *Cymbopogon flexuosus* (Nees ex Steud.) W.Watson | C306 |
|  | *Cynodon dactylon* (L.) Pers. | C307 |
|  | *Dactyloctenium aegyptium* (L.) Willd | C308 |
|  | *Digitaria sanguinalis* (L.) Scop. | C309 |
|  | *Eleusine indica* (L.) Gaertn. | C310/10074 |
|  | *Polypogon monspeliensis* (L.) Desf. | C311/10160 |
|  | *Saccharum officinarum* L. | C312 |
|  | *Setaria viridis* (L.) P.Beauv | C313 |
|  | *Bambusa balcooa* Roxb. | C314 |
|  | *Bambusa nutans* Wall. ex Munro | C315 |
|  | *Thysanolaena latifolia* (Roxb. ex Hornem.) Honda | C316/10117 |
| Polygonaceae | *Persicaria chinensis* (L.) H. Gross | C317/10152 |
|  | *Persicaria hydropiper* (L.) Delarbre | C318 |
|  | *Persicaria maculosa* Gray | C319/10106 |
| Pontederiaceae | *Eichhornia crassipes* (Mart.) Solms | C320 |
| Primulaceae | *Maesa indica* (Roxb.) DC. | C321 |
| Proteaceae | *Grevillea robusta* A.Cunn. ex R.Br. | C322/10116 |
| Rhamnaceae | *Ziziphus mauritiana* Lam. | C323/10122 |
| Rosaceae | *Rosa* sp. | C324 |
| Rubiaceae | *Ixora coccinea* L. | C325 |
|  | *Mussaenda philippica* A.Rich. | C326 |
|  | *Mussaenda roxburghii* Hook.f. | C327 |
|  | *Neolamarckia cadamba* (Roxb.) Bosser | C328/10096 |
|  | *Paederia foetida* L. | C329 |
|  | *Aegle marmelos* (L.) Correa | C330/10062 |
|  | *Citrus aurantiifolia* (Christm.) Swingle | C331 |
|  | *Citrus × aurantium* L*.* | C332 |
|  | *Citrus reticulata* Blanco. | C333 |
|  | *Citrus sinensis* (L.) Osbeck | C334 |
|  | *Murraya koenigii* (L.) Spreng. | C335/10063 |
|  | *Murraya paniculata* (L.) Jack | C336 |
|  | *Zanthoxylum rhetsa* DC. | C337 |
| Saliacaceae | *Populus deltoides* Marshall | C338/10094 |
|  | *Salix tetrasperma* Roxb. | C339 |
| Sapotaceae | *Mimusops elengi* L. | C340 |
| Saururaceae | *Houttuynia cordata* Thunb. | C341/10138 |
| Scrophulariaceae | *Bacopa monnieri* (L.) Wettst. | C342 |
| Simaroubaceae | *Ailanthus integrifolia* Lam. | C343 |
| Solanaceae | *Capsicum annum* L. | C344 |
|  | *Cestrum nocturnum* L. | C345 |
|  | *Datura metel* L. | C346 |
|  | *Lycopersicon esculentum* Mill. | C347 |
|  | *Physalis angulata* L. | C348/10109 |
|  | *Solanum abancayense* Ochoa | C349 |
| Thymelaeaceae | *Aquilaria malaccensis* Lam. | C350 |
| Tiliaceae | *Corchorus olitorius* L. | C351 |
| Ulmaceae | *Trema orientalis* (L.) Blume | C352 |
| Urticaceae | *Boehmeria allophylla* W.T.Wang | C353/10151 |
|  | *Pouzolzia hirta* Blume ex Hassk*.* | C354/10140 |
|  | *Dendrocnide sinuata* (Blume) Chew | C355 |
| Viraceae | *Leea aequata* L. | C356/10147 |
| Xanthorrhoeaceae | *Aloe vera* (L.) Burm.f. | C357 |
| Zingiberaceae | *Curcuma aeruginosa* Roxb. | C358 |
|  | *Curcuma longa* L. | C359/10081 |
|  | *Zingiber officinale* Roscoe. | C360/10082 |
